# Viruses as co-factors for the initiation or exacerbation of lung fibrosis

**DOI:** 10.1186/1755-1536-1-2

**Published:** 2008-10-13

**Authors:** Kevin M Vannella, Bethany B Moore

**Affiliations:** 1 Immunology Graduate Program, University of Michigan Medical School, Ann Arbor, MI 48109, USA; 2 Department of Internal Medicine, University of Michigan Medical School, Ann Arbor, MI 48109, USA

## Abstract

Idiopathic pulmonary fibrosis (IPF) remains exactly that. The disease originates from an unknown cause, and little is known about the mechanisms of pathogenesis. While the disease is likely multi-factorial, evidence is accumulating to implicate viruses as co-factors (either as initiating or exacerbating agents) of fibrotic lung disease. This review summarizes the available clinical and experimental observations that form the basis for the hypothesis that viral infections may augment fibrotic responses. We review the data suggesting a link between hepatitis C virus, adenovirus, human cytomegalovirus and, in particular, the Epstein-Barr gammaherpesvirus, in IPF. In addition, we highlight the recent associations made between gammaherpesvirus infection and lung fibrosis in horses and discuss the various murine models that have been used to investigate the contribution of gammaherpesviruses to fibrotic progression. We review the work demonstrating that gammaherpesvirus infection of Th2-biased mice leads to multi-organ fibrosis and highlight studies showing that gammaherpesviral infections of mice either pre- or post-fibrotic challenge can augment the development of fibrosis. Finally, we discuss potential mechanisms whereby viral infections may amplify the development of fibrosis. While none of these studies prove causality, we believe the evidence suggests that viral infections should be considered as potential initiators or exacerbating agents in at least some cases of IPF and thereby justify further study.

## Background

Idiopathic pulmonary fibrosis (IPF) is a progressive interstitial lung disease that severely compromises pulmonary function [1]. IPF likely results from an abnormal healing response to injury of the alveolar surface, and development of the disease is characterized by fibroblast hyperplasia and progressive collagen deposition that effaces normal lung tissue [2]. The median survival time for patients with IPF is 3 years from the time of diagnosis, and there is currently no effective treatment [3].

Fibrotic lung disease likely results from an inciting injurious event within the lung. Although the precise temporal sequence of events and mechanisms of disease are not understood, several common pathobiological characteristics are recognized. These include damage and loss of type I alveolar epithelial cells followed by hyperplastic expansion of type II cells [4]; variable chronic inflammatory cell infiltration [5]; a predominant T helper (Th)2 cytokine profile [6]; induction of pro-inflammatory cytokines, such as interleukin (IL)-8 and tumor necrosis factor (TNF)α [7,8]; induction of fibroblast growth factors, such as basic fibroblast growth factor and platelet-derived growth factor [9]; induction of differentiation molecules, such as transforming growth factor (TGF)-β1 [6,10]; an altered fibroblast phenotype characterized by exuberant proliferation and the transition to α-smooth muscle actin-positive myofibroblasts [11]; excessive deposition of extracellular matrix proteins [12]; derangements in eicosanoid synthesis, including increased leukotriene synthesis and diminished prostaglandin production [13,14]; diminished activation of plasminogen and altered coagulation cascades [15,16]; and recruitment of bone-marrow derived fibrocytes [17-19].

Despite ongoing research driven by the need for therapy, the initiating or injurious agents are unknown, and it is not understood why the fibrosis is dysregulated and progressive [20]. It is likely that the disease initiates with some form of alveolar epithelial cell injury. This could be in the form of inhaled toxins or due to genetic abnormalities, such as mutations in telomerase or surfactant protein C [21-25], but more research is needed to fully understand the etiology of lung fibrosis. The natural history of the disease can take at least two forms. Patients can experience a gradually progressive disease, characterized by steady worsening of symptoms, lung function and gas exchange [26,27] or they can experience an acute respiratory deterioration termed an acute exacerbation noted by rapid worsening of symptoms over a short time frame (usually less than 1 month) [28]. Acute exacerbations can have extremely high mortality rates (reviewed in [28]). Both the development of IPF and the onset of acute exacerbations are idiopathic but may involve toxic exposures, genetics, aspiration, disordered coagulation and complications of comorbidities [28]. An emerging hypothesis is that occult infections may play a pathogenic role as co-factors for the development of IPF or acute exacerbations. It is possible that the chronic presence of an inflammatory agent like a virus in a genetically susceptible host disrupts the normal healing response, thus making the lung highly susceptible to a separate injurious trigger. Viruses are intriguing candidates for a role in IPF because of their ubiquitous incidence in humans and because of the nature of their lifecycle. Some viruses exist as an antigenic stimulant in the epithelial cells of the lung in an actively replicating and potentially injurious lytic phase while other viruses persist in a latent phase for an entire lifetime. It is interesting to note that some viruses, such as Epstein-Barr virus (EBV), which has been linked to IPF, are known to infect most people at some point in their life [29]. This raises the interesting question of why some people may develop IPF in response to this infection while others may not. Clearly, there is no easy answer, but it is likely that differential host responses to the virus may alter the pathogenesis. For instance, latent EBV infection is most often found in B cells [29]; however, in patients with IPF, EBV can be found in lung tissue, including epithelial cells [30,31]. Alternatively, stress, drug exposures or immunodeficiency may be responsible for viral reactivation in some patients, but not others. In this review, we discuss the available evidence that hepatitis C, adenovirus and cytomegalovirus infections are found in association with IPF. By far the most compelling evidence for a viral co-factor in IPF comes from studies of the association between the gammaherpesvirus, EBV, and IPF. The data regarding the role of EBV in IPF will be discussed last and will then be followed by a discussion of animal models that demonstrate a pro-fibrotic role for gammaherpesvirus infections.

### Human studies that have suggested a link between IPF and particular viral infections

#### Hepatitis C

Two studies have suggested a link between infection with the hepatitis C virus (HCV) and IPF. HCV is a small, enveloped, positive-sense single-stranded RNA virus in the Flavivirus family [32]. Replication within hepatocytes causes a form of hepatitis, but the virus may be able to enter other cell types [33]. Ueda *et al*. [34] were able to show a significant difference between the percentage of Japanese IPF patients who had serum antibodies to HCV (28.8%) and the corresponding percentage of control subjects (3.66%). A subsequent Italian study confirmed that 13% of patients with IPF were seropositive for HCV [35]. In a control group of 4,614 blood donors, the prevalence of HCV antibodies was lower (0.3%). However, in a control group of 130 patients with non-interstitial lung disease, HCV antibody prevalence was 6.1%. While this was significantly higher than the blood donor group, the incidence of HCV in patients with non-interstitial lung disease and IPF were not statistically different. It should also be noted that a British study failed to find an association between IPF and HCV [36]. One possible explanation for these findings is that there may be geographical differences in the prevalence of HCV infection, as the infection is more commonly seen in Japan and Mediterranean countries than it is in northern Europe [35]. Given that HCV is not known to replicate in the lung, it is not clear whether these associations suggest that HCV is pathogenic in IPF, or rather if they indicate that IPF patients develop HCV cross-reactive antibodies. Thus, further research in this area will be needed.

#### Adenovirus

Human adenoviruses have been suggested as etiological co-factors in the progression of interstitial lung disease [37-39]. Adenoviruses are medium-sized, non-enveloped, icosahedral, double-stranded DNA viruses. They are relatively resistant to chemical and physical agents, and as a result, they can remain infectious outside of the body for extended periods of time. Adenovirus infections typically cause respiratory symptoms and can be shed for long periods of time post-infection [37]. Kuwano *et al*. [38] examined 19 patients with IPF, 10 patients with interstitial pneumonia associated with collagen vascular disease, and 20 patients with sarcoidosis using nested PCR and *in situ *hybridization for the adenovirus gene product E1A. E1A DNA was present in 3 out of 19 (16%) cases of IPF, in 5 of 10 (50%) cases of interstitial pneumonia associated with collagen vascular disease, and in 2 of 20 (10%) cases of sarcoidosis [38]. While these data are not suggestive of a correlation between adenovirus infection and pulmonary fibrosis, Kuwano *et al*. found that the incidence of E1A DNA was considerably higher in patients who had been treated with corticosteroids (67%) compared to those patients left untreated (10%). This finding raises the interesting possibility that corticosteroids, a common therapy for IPF, may make patients more susceptible to adenovirus infection or reactivation from latency. However, studies investigating the titer of anti-adenoviral IgG in IPF patients have failed to demonstrate an elevation above normal [40]. Despite this, it is important to note that E1A has been shown to upregulate production of the pro-fibrotic mediator, TGF-β, and to induce lung epithelial cells to express mesenchymal markers [41]. This mechanism has been implicated in the architectural remodeling that occurs in chronic obstructive pulmonary disease. It is possible that a similar mechanism could contribute to extracellular matrix deposition and remodeling in those IPF patients who may also have adenovirus infections.

It is also conceivable that adenovirus infections could serve as exacerbating agents for patients with established lung fibrosis, although it is difficult to determine the frequency of this happening from published clinical literature. Furthermore, recent studies using an animal model of fluorescein isothiocyante (FITC)-induced fibrosis were unable to demonstrate significant exacerbation of FITC-fibrosis within the first 7 days post-mouse adenoviral infection [42]. One note of caution in the interpretation of these experiments, however, is that human and mouse adenoviruses do show different tropisms, with human adenoviruses being predominantly respiratory pathogens. Interestingly, in studies using the same murine model, a gammaherpesvirus was able to augment FITC-induced fibrosis (see below). Whether the difference in the ability of mouse adenovirus and mouse gammaherpesvirus to exacerbate FITC-fibrosis represents differences in cell tropisms, inflammatory response or mediators released is unknown.

#### Human cytomegalovirus

Human cytomegalovirus (HCMV), a betaherpesvirus, is a widespread opportunistic pathogen that persists in healthy individuals but normally only causes clinical manifestations in immune-compromised individuals [43]. HCMV infects the respiratory tract, and it has been evaluated with regards to IPF. Dworniczak *et al*. [44] studied 16 patients, newly diagnosed with IPF and never treated, compared to 16 adult healthy volunteers. HCMV DNA copy number in broncho-alveolar lavage (BAL) cells, blood leukocytes, and serum was calculated by real-time PCR, and the prevalence of the HCMV DNA positive subjects in the patient group (75%) did not differ significantly from the prevalence of positive subjects in the control group (69%). IPF patients did show significantly higher DNA copy numbers in their blood compared to controls, however [44]. Also, the viral copy number in the BAL cells of both IPF patients and healthy volunteers was elevated relative to respective viral copy numbers in blood leukocytes, suggesting an important role for the lungs (perhaps as a viral reservoir) in the pathobiology of HCMV. Consistent with this idea, a subsequent study by Tang *et al*. [45] did note higher levels of HCMV DNA in IPF lung tissue compared to control samples. Similarly, in a study by Yonemaru *et al*. [40], HCMV IgG and complement fixation titers were found to be elevated in the serum of patients with IPF when compared to several other disease-specific controls. In a retrospective study of lung transplant recipients, 102 patients were screened by urine test for evidence of HCMV infection on the day of transplant. Only five patients were found to be HMCV+ prior to transplant, and all five of the patients were IPF patients [46]. Despite testing positive, none of these five patients exhibited symptoms of HCMV disease, suggesting that viral infections in this population can be occult. The increased incidence of HCMV infection prior to transplant correlated with an increased risk of HCMV infection post-transplant as well. In sum, there is evidence that suggests an association between the incidence of HCMV infection and the incidence of IPF, but a mechanism by which HCMV may affect fibrosis remains to be elucidated.

#### Epstein-Barr virus

The virus that has been associated most strongly with IPF is EBV. EBV is a gammaherpesvirus that is present in all populations, infecting more than 95% of humans within the first decades of life [47]. An association between EBV infection and IPF was first established when elevated levels of immunoglobulins A and G against EBV antigens were measured in a serological study of 13 patients with IPF [48]. In contrast, 12 patients with interstitial lung disease of known cause had normal EBV serological profiles. This finding led to further research on EBV in the context of IPF. An immunohistochemical study indicated that EBV replicates within epithelial cells of the lower respiratory tract in IPF patients [49]. Consequently, Stewart *et al. *[50] sought to confirm the presence of EBV DNA in the lung tissue of IPF patients using PCR. They found that EBV was present in the lungs of patients with IPF at a significantly higher percentage (48%) than in the lungs of control subjects (14%).

A couple of studies have associated the presence of active and latent EBV markers with IPF. Kelly *et al*. [51] investigated the occurrence of productive EBV replication by analyzing for the presence of an EBV gene rearrangement termed WZhet; 61% of EBV DNA-postive lung tissue biopsies from IPF patients were positive for WZhet. Buffy coat analysis for WZhet was positive in 16 of 27 IPF patients compared to none of 32 lung transplant recipients and 1 of 24 normal blood donors. Tsukamoto *et al. *[52] then determined that the presence of EBV latent membrane protein 1 (LMP1) is linked with more rapid disease progression. From a group of 29 patients, they found that patients positive for LMP1 died more quickly than patients who tested negative for EBV.

It should be noted that not all studies have found an association between EBV and IPF. In 1997, Wangoo *et al*. [53] published findings contrary to previous reports when they did not detect any EBV DNA in the lungs of IPF patients. Also, in 2005, an Italian study by Zamo *et al*. [54] failed to find evidence of either EBV or human herpesvirus (HHV)-8 DNA in their tissue banks of IPF samples. Whether these discrepancies reflect geographical distribution, technical sensitivities, or disease heterogeneity is still unclear.

Although EBV had been detected with more frequency in the lungs of IPF patients than in the lungs of control patients in most previous studies, many members of each IPF cohort analyzed did not test positive for EBV infection at all. Tang *et al. *[45] went on to test the hypothesis that at least one herpesvirus could be detected in the lungs of all IPF patients. They identified one or more of four herpesviruses – EBV, HCMV, HHV-7, and HHV-8 – in 32 of 33 patients with IPF and in 9 of 25 controls. They found two or more herpesviruses in 19 of 33 IPF patients and in 2 of 25 controls. These data strongly support the notion that at least one herpesviral infection accompanies the development of IPF.

Tang *et al. *drew other conclusions from their study that suggest susceptibility to viral infection and IPF depends on a genetic or acquired predisposition. Co-infection occurred more frequently in patients with the sporadic form of IPF compared to those with the familial form. Familial IPF is characterized by the incidence of IPF in two or more members of an immediate family [55]. This led the authors to suggest that a patient with familial IPF may require less viral influence to trigger a progressive fibrotic response than a patient with sporadic IPF. In addition, Tang *et al*. note that the increased frequency of HHV-8 in the lungs of the IPF cohort is particularly interesting. In the United States, HHV-8 infection is predominantly found in patients with HIV infection and Kaposi's sarcoma [56], and all of the subjects tested negative for HIV in this study.

Collectively, the analyses of IPF lung tissue chronicled above create a rationale to study the association between viral infections and the occurrence of IPF, but do not provide evidence for a causal relationship between viruses and IPF. Demonstrating causation in humans requires detection of a virus in the lungs prior to clinical manifestations of IPF (which is clinically implausible) or evidence that an anti-viral therapy confers anti-fibrotic effects. The latter has been attempted with some success in a limited number of case studies, but no large trials have been conducted to date [45,57]. While at least four different viruses have been correlated with IPF, the most striking observations have linked EBV infection of lung tissue with the presence of IPF. However, we want to again stress that the pathogenesis of IPF is complex and multifactorial. In reality, IPF is likely a spectrum of diseases that result from a variety of genetic abnormalities and/or environmental factors. As mentioned above, the data regarding the association of viruses, even EBV, with IPF are controversial. What is particularly intriguing to us, however, is that these clinical observations are somewhat strengthened by emerging evidence in animal models that demonstrate that gammaherpesvirus infections can be linked to the development or the exacerbation of experimentally induced fibrosis. These animal studies will be highlighted below.

### Animal studies that support a role for gammaherpesvirus infections as initiators or co-factors for the development of fibrosis

#### Naturally occurring cases of gammaherpesvirus infection and fibrosis

Pulmonary fibrosis has been reported to occur in both cats and horses [58-62]. Interestingly, recent studies of both horses and a single donkey have reported pulmonary interstitial fibrosis associated with herpesvirus-associated pneumonia [59,63]. It is interesting to note that infection with equine herpesvirus-5, a gammaherpesvirus, was detected by PCR in 19/24 (79.2%) horses affected with interstitial fibrosis whereas only 2/23 (8.7%) of control horses showed evidence of infection [59]. While these data do not prove causality, the association between gammaherpesviruses and fibrosis in both horses and humans is striking.

#### Murine modeling to demonstrate a causal role for gammaherpesvirus infection in the augmentation of fibrosis

Murine models of pulmonary fibrosis have enabled identification of pathogenic cells and mediators that are believed to be important in human fibrotic disease, and they have facilitated further exploration of a pathogenic role for viruses in humans [64]. The human herpesviruses identified to be prevalent in IPF lung tissue have limited infection capability in mice, however. Thus, investigators have utilized a natural murine pathogen called murine gammaherpesvirus (MHV)-68 that has been characterized as genetically and biologically closely related to human gammaherpesviruses. The genome of MHV-68 is largely colinear with EBV and HHV-8, and there is evidence that both murine and human gammaherpesviruses infect the respiratory tract and can persist in B cells as well as lung epithelial cells [65-69].

#### MHV-68 infection prior to bleomycin administration worsens fibrosis

**Figure 1:**
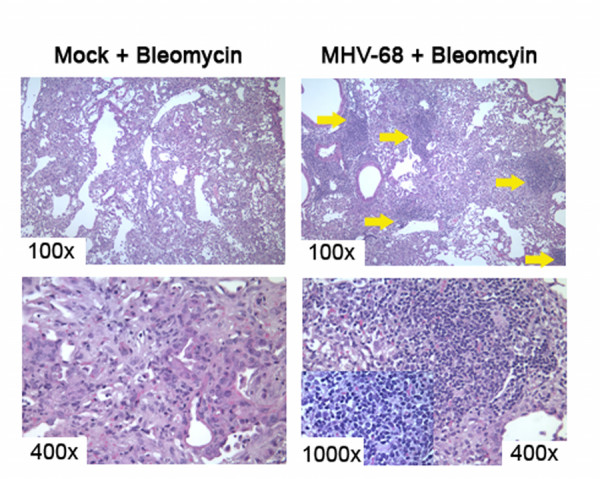
Herpesviral infections change the nature of the inflammatory response to subsequent fibrotic stimuli. Mice were given 5 × 10^4 ^pfu MHV-68 or saline 14 days prior to the instillation of bleomycin as a fibrotic stimulus. Mice were euthanized and lungs were prepared for histology 21 days post-bleomycin. Panels on the left-hand side represent mice pre-treated with saline, and then challenged with bleomcyin. Panels on the right-hand side represent mice infected with MHV-68 prior to the bleomcyin challenge. MHV-68 was latent at the time of bleomycin inoculation. Viral pre-infection causes increased numbers of inflammatory cells to enter the lung during the subsequent fibrotic response. We have verified this with collagenase digests and cell counts as well (not shown). It is particularly interesting that the virally infected mice show focal clusters of mononuclear cells (yellow arrows) that are not seen in the mice challenged with bleomycin alone. The inset in the lower right panel is a 1,000× magnification of one of these mononuclear foci. Based on morphology, these cells are likely leukocytes.

In 2002, Lok *et al. *[70] used MHV-68 to demonstrate that gammaherpesviruses could serve as a cofactor in the development of pulmonary fibrosis. BALB/c mice infected intranasally with MHV-68 one week prior to intratracheal administration with the fibrotic stimulant bleomycin later developed pulmonary fibrosis even though BALB/c mice are normally resistant to bleomycin. Mice infected with MHV-68 but not challenged with bleomycin did not develop fibrosis. The gammaherpesvirus infection alone was not sufficient to cause fibrosis, but Lok *et al. *proposed that a viral infection made the previously protected lungs susceptible to fibrotic disease upon the event of an exogenous injury. These results are enticing, but it should be noted that the bleomycin was delivered during the peak of lytic viral infection. It is difficult to infer from these studies whether chronic latent infection with MHV-68 might also predispose the lung to subsequent fibrotic responses. Also, the mechanism(s) for how MHV-68 infection augmented the subsequent fibrotic response to bleomycin were not defined. This is an area of active research in our laboratory. We have determined that MHV-68 infection is latent in the lung by day 14 post-infection [42]. Mice given MHV-68 14 days prior to the administration of bleomycin and harvested 21 days post-bleomcyin show a different pattern of inflammation and fibrosis than is noted in mice mock infected prior to bleomycin administration (Figure 1). Of note, focal clusters of mononuclear inflammatory leukocytes are seen in the setting of viral infection. Thus, it is likely that latent viral infections alter the inflammatory response that occurs in response to a secondary fibrotic stimulus.

#### MHV-68 infection in Th2-biased mice causes multi-organ fibrosis

Ebrahimi *et al*. [71] showed that MHV-68 causes fibrosis in interferon (IFN)γR-/- mice. IFN-γ is a Th1 cytokine with anti-viral and anti-fibrogenic properties. It down-regulates the expression of both type I and type III collagens and fibronectin [72-75]. The cytokine profile of IFNγR-/- mice is Th2-biased, resulting in a cytokine imbalance similar to that observed in the lungs of IPF patients [76]. This study suggests that a herpesvirus infection delivered to lungs skewed towards a profibrotic cytokine environment is sufficient for fibrogenesis. In this case, the fibrogenesis was not limited to the lung only. The mice developed multi-organ fibrosis (liver, lung, spleen and lymph nodes). The development of multi-organ fibrosis correlated with an overproduction of pro-fibrotic mediators such as TNF-α, TNF-β, IL-1β, TGF-β1, lymphotactin, and macrophage inflammatory protein-1β (MIP-1β). These mediators were elevated on day 14 after infection whereas the anti-fibrotic chemokines CXCL9 (CXC chemokine ligand 9) and CXCL10 were significantly reduced. The authors noted that MHV-68 gene expression may have influenced the cytokine imbalance. These results are fascinating in light of the fact that MHV-68 infection in wild-type mice shows no signs of causing multi-organ fibrosis and suggest that the outcome of viral infection may be critically dependent on the cytokine milieu at the time of infection.

Mora *et al*. [57] extended studies in the IFNγR-/- model of herpesvirus-induced fibrosis by characterizing the disease seen in the lungs and examining possible pathogenic effects of the virus that contribute to fibrosis. They showed that MHV-68 induces epithelial damage and inflammatory responses leading to alveolar remodeling and ultimately to unresolving progressive interstitial fibrosis resembling human IPF. More recently, Mora *et al*. [77] published data implicating alveolar macrophages as integral profibrotic effectors in IFNγR-/- mice. These studies suggested that alveolar macrophages were chronically recruited to areas of epithelial hyperplasia and fibrosis and that these alveolar macrophages displayed signs of alternative activation, a process known to be driven by Th2 cytokines. The alternatively activated macrophages express arginase. Arginase metabolism of L-arginine to L-ornithine, L-proline, and polyamine promotes fibroblast proliferation, collagen production, and, ultimately, fibrosis. In addition, there is evidence that microvascular injury has a role in the pathogenesis of IPF [78], and Mora *et al*. [57] detected viral-induced vasculitis accompanied by red blood cell extravasation compatible with hemorrhage. With regards to viral-induced vasculitis, it is interesting that Magro *et al*. [78], who proposed microvascular injury as a pathogenic mechanism for IPF, also found evidence of CMV and parvovirus B19 infection in IPF patients. In fact, Magro *et al*. [78] speculated that endotheliotropic viral infections, such as CMV and B19, may be precursors for the microvascular injury that is noted in IPF [78]. Similar mechanisms may be responsible for the MHV-68-induced damage noted in the studies by Mora *et al. *MHV-68 has previously been reported to cause vascular damage [79]. Finally, Mora *et al*. [57] noted enhanced expression of the profibrotic cytokine TGF-β1 in the MHV-68-infected IFNγR-/- mice, and it is likely that this pro-fibrotic cytokine may make a significant contribution to the fibrosis in this model. In wild-type mice, however, IFN-γ signaling would be expected to inhibit transcription of the TGF-β gene [80].

The authors attribute some of the TGF-β1 dysregulation to epithelial cell damage. Interestingly, type II alveolar cells are a target of MHV-68, and Mora *et al*. [57] speculate that epithelial cell infection and injury may be triggering surfactant abnormalities as well as dysregulated epithelial cell repair. An association between the frequency of polymorphic variants of surfactants and IPF has been documented previously [81]. Furthermore, another series of studies on IPF has found some evidence that a link between EBV and p53 expression leads to modifications in epithelial cell repair and apoptosis [82,83]. Flano *et al*. [84] have reported chronic low level reactivation of MHV-68 in the lung. It is possible that reactivation is a repetitive trigger in the setting of the IFNγR-/- mice that contributes to the progressive disruption of lung epithelial cells and unresolving fibrosis. In support of this, preventing chronic reactivation of MHV-68 to the lytic phase through the use of anti-viral drugs reduces fibrosis in IFN-γR-/- mice [84,85]. Table 1 summarizes potential mechanisms whereby preceding viral infection may predispose the host to the development of fibrosis.

**Table 1:** Potential mechanisms to explain how viral infections may predispose the host to develop fibrosis

Lytic infections may kill lung epithelial cells
Latent infections may alter the phenotype (proliferation, apoptosis or mediator secretion) of various lung cells (for example, epithelial and mesenchymal cells)
Persistent viruses may provide repeated insults with reactivation
Infection may increase the production of pro-fibrotic mediators (for example, TGF-β) or diminish the production of anti-fibrotic mediators
Induction of epithelial to mesenchymal transition
Induction of chemokines and fibrocyte recruitment
Surfactant abnormalities
Enhanced inflammation
Alteration of p53 function
Microvascular injury

#### MHV-68 as an exacerbating agent for established fibrosis

While most IPF patients have a slow, progressive disease, some patients have an acute deterioration in function that carries a poor prognosis. In the placebo arm of a study of 32 patients who died from IPF-related causes, Martinez *et al*. [86] reported that 47% suffered an acute deterioration, and 27% of the acute deteriorations were associated with infection. We were recently able to model acute exacerbations of fibrosis in a murine model of FITC-induced pulmonary fibrosis. Wild-type mice infected with MHV-68 after the establishment of fibrosis (day 14) developed a significantly worse fibrotic response than fibrotic mice that were mock-infected with saline [42]. This finding is especially interesting in light of the work done in Th2-biased mice discussed above because MHV-68 exacerbation of fibrosis in wild-type mice occurred despite a strong Th1-biased anti-viral immune response. In fact, MHV-68 was able to exacerbate FITC-induced fibrosis even in Th2-deficient (IL-4 and IL-13-/-) mice [42]. In these experiments looking at exacerbation of established fibrosis, MHV-68 infection was lytic. The pro-fibrotic actions of MHV-68 may vary greatly depending on whether the infection precedes or follows the fibrotic stimulus, and the contribution of Th2 cytokines to the pathogenesis may vary as well depending on the timing of infection.

The recruitment of fibrocytes to the lung was also associated with viral exacerbation of FITC-induced fibrosis [42]. Fibrocytes are bone marrow-derived cells that share the characteristics of both leukocytes and mesenchymal cells and are defined by the co-expression of CD45 and collagen 1 [17]. Fibrocytes migrate in response to chemokine signals and likely contribute to fibrogenesis both via differentiation into myofibroblasts as well as through the paracrine secretion of pro-fibrotic mediators [18,19,87-89]. If MHV-68 infection is able to recruit fibrocytes, then this mechanism may be able to explain the enhancement of fibrosis noted by Lok *et al*. [70] when the infection preceded the administration of bleomycin by 7 days. In fact, if MHV-68 infection in the lung is associated with prolonged recruitment of fibrocytes even after the virus has established latency, this could explain why herpesviral infections may predispose persons to an enhanced fibrotic response upon a second challenge. It may also explain, in part, the altered inflammatory responses noted when mice latently infected with MHV-68 are challenged with a fibrotic stimulus (Figure 1). We have gathered preliminary data in our lab that suggest that fibrocyte accumulation in the lung is elevated for at least 30 days post-MHV-68 infection (unpublished observation). Thus, this will be an area of active future exploration. It is likely that viral infections alter epithelial cell function, cytokine profiles and inflammatory cell accumulation to promote fibrosis. Table 2 summarizes potential mechanisms to explain the ability of a viral infection to exacerbate existing pulmonary fibrosis.

**Table 2:** Potential mechanisms for viral exacerbation of IPF

Th2 environment in fibrotic lungs may limit viral clearance
Epithelial cells in fibrotic lungs may be unable to effectively replace epithelial cells damaged by lytic infection
Increased chemokines may recruit and activate fibrocytes
Pro-inflammatory mediators secreted in response to infection may have pro-fibrotic effects
Steroid and immunosuppressive therapy may predispose the host for increased viral infection or reactivation
Microvascular injury

## Conclusion

**Figure 2:**
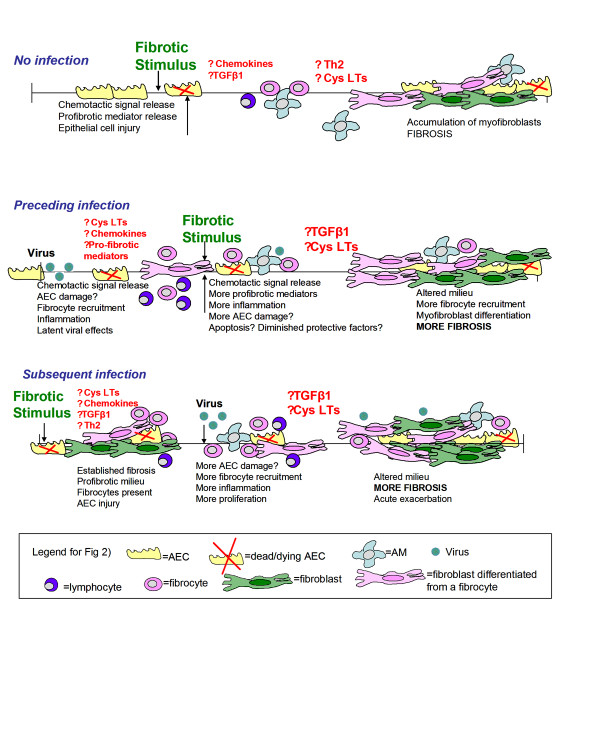
Schematic representation depicting potential mechanisms involved in viral augmentation of fibrosis. Viral infection may precede the fibrotic insult or occur subsequent to the fibrotic challenge. In both cases, recruitment of inflammatory cells, including fibrocytes, increases in pro-fibrotic mediator production and epithelial cell injury may play important roles.

The studies with MHV-68 discussed here can only suggest that similar human viruses found in the lungs of IPF patients have a pathogenic role in fibrosis. Given that fibrosis in humans is progressive, it seems important to better understand the chronic reactivation that can occur during herpesviral infections. It is possible that repeated activation may lead to repeated rounds of epithelial cell damage, cytokine release and fibrocyte recruitment. Additionally, it will be important to determine whether long-term latent MHV-68 infection of naïve mice augments the production of profibrotic factors and whether the same is true in EBV-infected human lungs. A latent infection does not appear to create a Th2 bias equal to that of IFN-γR-/- mice, but any bias may predispose a latently infected individual to a fibrotic trigger from an unrelated factor. It will also be helpful to more carefully study the cell types that harbor long-standing viral infection because the prolonged injury caused to such a cell type is likely responsible for increasing susceptibility to fibrosis. Flano *et al*. [84] found that long-term latency in the lung is maintained primarily in B cells, but they also showed that MHV-68 maintains persistent replication in the lung at least 3 months after infection, suggesting that cell types besides B cells may be re-infected. In fact, Stewart *et al*. [68] have suggested that lung epithelial cells may be long-term reservoirs of persistent viral infection. Epigenetic changes caused by infection should also be explored. Genetic deficiencies in leukotriene production and expression of the CCR2 receptor have been shown to be protective against fibrosis in animal models independent of viral infection [90,91]. As viral infections are known to induce the production of both leukotrienes and chemokines, it may be important to understand how significant such mediators are to a virus-induced predisposition to fibrosis. It is also possible that the additional inflammatory cells recruited by the lytic or latent virus may alter the fibrotic milieu. In summary, evidence from both clinical studies and animal models suggest that viruses, especially gammaherpesviruses, may be co-factors for the development or exacerbation of lung fibrosis and have suggested that fibrocyte recruitment may be one pathogenic mechanism. Figure 2 provides a schematic representation that depicts potential mechanisms whereby viral infections (either preceding the fibrotic response or subsequent to it) may enhance fibrotic outcomes. Through the use of new animal models, researchers should be able to elucidate additional pathogenic mechanisms with the hope of finding new therapies.

## Competing interests

The authors declare that they have no competing interests.

## Authors' contributions

KMV performed literature searches and wrote the bulk of the review. BBM reviewed and edited the article for content and clarity.
